# Intracisternally Injected L-Proline Activates Hypothalamic Supraoptic, but Not Paraventricular, Vasopressin-Expressing Neurons in Conscious Rats

**DOI:** 10.4061/2011/230613

**Published:** 2011-10-16

**Authors:** Yumi Takemoto

**Affiliations:** Department of Neurophysiology, Graduate School of Biomedical Sciences, Hiroshima University, Kasumi 1-2-3, Minami-ku, Hiroshima 734-8551, Japan

## Abstract

When injected into specific rat brain regions, the neurotransmitter candidate L-proline produces various cardiovascular changes through ionotropic excitatory amino acid receptors. The present study used an immunohistochemical double-labeling approach to determine whether intracisternally injected L-proline in freely moving rats, which increases blood pressure, activates hypothalamic vasopressin-expressing neurons and ventral medullary tyrosine-hydroxylase- (TH-) containing neurons. Following injection of L-proline, the number of activated hypothalamic neurons that coexpressed vasopressin and c-Fos was much greater in the supraoptic nucleus (SON) than in the paraventricular nucleus (PVN) of rats with increased blood pressure. The number of activated TH-containing neurons was significantly greater following L-proline treatment than following control injections of artificial cerebrospinal fluid (ACSF). These results clearly demonstrate that intracisternally injected L-proline activates hypothalamic supraoptic, but not paraventricular, vasopressin-expressing neurons and medullary TH-containing (A1/C1) neurons in freely moving rats.

## 1. Introduction

The nonessential imino acid L-proline has been proposed to be a neurotransmitter or neuromodulator of the central nervous system [1–3]. It produces various functional changes in animals, such as cardiovascular changes in rats [4–8] and sedation as well as hypnotic effects under stressful conditions in chicks [9, 10]. Intracisternal injections of L-proline, but not D-proline, have been shown to cause an increase in blood pressure in freely moving rats in a dose-dependent manner via ionotropic excitatory amino acid receptors in the brain [4, 5, 11]. This hypertensive response to centrally administered L-proline can be almost inhibited by intravenous preinjection of a vasopressin V1 receptor antagonist alone and augmented in ganglionic blocking rats where the augmented response was completely abolished by the additional vasopressin receptor antagonist [11], suggesting that the L-proline-induced pressor response could be mainly mediated by the release of hypothalamic vasopressin into the blood stream. Previous studies have shown that intracisternally injected dye robustly stains the medullary surface [12, 13]. These results suggest that intracisternally injected L-proline might diffuse and reach the medullary A1 catecholamine neurons, which send their terminals to vasopressin-expressing neurons in both the paraventricular nucleus (PVN) and the supraoptic nucleus (SON) of the hypothalamus [14]. The goal of this study was to determine the identity of the L-proline-responsive cells in freely moving rats using double immunohistochemical detection of c-Fos protein as a marker of neuronal activation and vasopressin or the enzyme tyrosine hydroxylase (TH), a catecholamine marker. 

Brief reports of this work have appeared in abstract form [15, 16].

## 2. Methods

All protocols and surgical procedures used in the current study were performed in accordance with the Guiding Principles for the Care and Use of Animals approved by the Council of the Physiological Society of Japan, the guidelines of the Committee of Animal Experimentation, Hiroshima University, and the Committee on Research Facilities for Laboratory Animal Science, Natural Science Center for Basic Research and Development (N-BARD), Hiroshima University.

### 2.1. Animal Care and Preparation

Eighteen male Wistar rats (300–350 g), which were cared for and handled by an experimenter for at least three weeks prior to surgery, were anesthetized with sodium pentobarbital (i.p., 50 mg/kg). Intracisternal polyethylene tubing was placed and fixed on the cranial bone with dental cement, as described previously [11]. Seven days after the first operation, the rat was reanesthetized with sodium pentobarbital (i.p., 50 mg/kg), and a polyethylene catheter (sp 10 fused to sp 19, Natsume, Japan) was placed in the terminal aorta to perform arterial pressure measurements, heart rate measurements, and drug administration. To reduce operation-induced stress, the protocol excluded the venous catheter insertion step used in previous studies [4, 5, 11]. Cages were placed in a room with a controlled 12 h light and 12 h dark cycles. Water and food pellets were supplied *ad libitum*.

### 2.2. Hemodynamic Measurements

The experiments with freely moving rats began 2 d after the final operation. The experiments were started in the morning between 8:00 and 10:00 AM in their home cages. The rat was given a resting period of at least 60 min while its arterial blood pressure and heart rate were monitored, as described previously [11]. These parameters were recorded by a pen-writing oscillograph following an injection of either intracisternal L-proline (10 *μ*L of 1 M L-proline) or artificial cerebrospinal fluid (ACSF; 15 *μ*L) for 90 min. Thereafter, Evans blue dye solution (10 *μ*L, 0.2%) was injected to examine the distribution of L-proline in the brain.

### 2.3. Brain Slice Preparation

Ten minutes after injecting the dye, sodium pentobarbital (50 mg/kg) was slowly infused intra-arterially. The abdominal aorta of the rat was blocked with a hemostat secured below the level of the thoracic aorta, and the upper body was perfused intracardially with isotonic saline containing heparin (10 units/ml, 20–40 mL), followed by fixative solution containing freshly prepared 4% paraformaldehyde buffered with sodium phosphate (200 mL, pH 7). After observing the dye distribution over the surface of the whole brain, each brain block, including the hypothalamus or medulla, was postfixed for 2 h. Coronal slices that were 50 *μ*m thick were made using a vibratome. Alternating sections were used for each immunohistochemical staining series. The sections were kept in sequential order using a custom-made 24-well culture plate with a mesh fiber sheet on the bottom.

### 2.4. Immunohistochemistry

Floating sections were pre-treated with 0.3% H_2_O_2_ in phosphate buffered saline (PBS) for 30 min, followed by incubation in blocking solution (3% bovine serum albumin and 0.3% triton X-100 in PBS, pH 7) for 90 min. The sections were incubated overnight with a rabbit polyclonal anti-c-Fos antibody (1 : 10,000, Calbiochem PC38, ex-Oncogene Ab-5) [17] diluted in blocking solution containing sodium azide (0.2%). The sections were incubated with a biotinylated goat anti-rabbit IgG secondary antibody diluted in blocking solution for 2 h, followed by incubation with horseradish peroxidase avidin D reagent diluted in PBS for 1 h. The staining was detected as a black complex following the reaction with 3,3′-diaminobenzidine tetrahydrochloride (DAB, Dojindo Laboratories, Japan) with 0.006% H_2_O_2_, 1% cobalt chloride, and 1% nickel ammonium sulfate [17]. To perform double staining, the sections were further incubated overnight with either a rabbit polyclonal antibody against vasopressin (1 : 17000, Chemicon AB 1565, which exhibited less than 1% cross-reactivity with oxytocin) or a rabbit polyclonal antibody against TH (1 : 2500, Protos Biotech Corp, New York, CA-101-bTHrab). In pilot experiments, each antibody was tested to confirm that it labeled cells with DAB in the appropriate neuronal groups, including the vasopressin-expressing neurons in the SON and PVN and TH-expressing neurons in the medulla [18–20]. A similar reaction was used for vasopressin or TH detection; however, only the DAB-H_2_O_2_ solution was used to produce a detectable brown product in the cytoplasm. Sections were washed thoroughly with PBS between each reaction. Following antibody detection, the sections were mounted on gelatinized glass slides, air-dried overnight, dehydrated, cleared with xylene solutions, mounted with Permount SP15 (Fisher Scientific), and sealed with a coverslip.

### 2.5. Counting of Labeled Cells

The sections were examined using a light microscope equipped with a mounted digital camera, and images were captured using a DP70 Image Analysis System (Olympus). The positively stained cells were manually counted from printed, magnified photographs. The corresponding region was kept under the microscope as a reference to adjust the focus on other vertical levels in the section if necessary. The counts were made by a researcher blinded to the treatment. When counting stained cells in the hypothalamus, a print of a highly magnified photograph (100x) was used for selected slices that had the densest staining at each nucleus level; these slices correspond to the middle photographs shown in Figure 2. For analysis of the frontal slices of the medulla, which have similar structures throughout the levels, photographs were first taken in sequential order at low magnification (20x), and then three slices −14.2 mm (the caudal tip of the area postrema), −13.6 mm, and −12.6 mm from Bregma were determined using a brainstem atlas [20]. After numbering all of the levels of the slices in the photographs, TH-positive cells with c-Fos expression in the medulla were counted at higher magnifications in the corresponding slices. The numbers of cells in two successive slices (doubled 50 *μ*m thickness = 100 *μ*m) were combined in every 0.2 mm spaced in Figure 5. The data shown in Figure 5 correspond to slices from more than four rats.

The results are expressed as means ± SE. The data were analyzed parametrically using grouped *t*-tests, repeated measures one-way analyses of variance (ANOVAs) followed by Dunnett's test, or repeated measures two-way ANOVAs followed by Scheffe's test. Several groups of data were also analyzed nonparametrically using the Wilcoxon rank sum test, as indicated. *P* values <0.05 were considered statistically significant.

## 3. Results and Discussion

The current immunohistochemical investigation combined with c-Fos detection as neuronal activation markers was performed to identify possible neurons in the hypothalamus and the medulla responding to L-proline injected into the cisterna magna in the freely moving rat. Microinjections of L-proline into the nucleus tractus solitarii [6] and the caudal ventrolateral medulla [7, 8], which are nuclei related to cardiovascular control, produced a depressor response via ionotropic excitatory amino acid receptors in urethane-anesthetized rats. However, anesthetized rats did not respond to intracisternally injected L-proline in pilot experiments. General anesthesia modifies functions of ion channels mainly for chloride and potassium to induce loss of consciousness in animals [21]. It means a possible antagonized function of anesthesia against original responses to L-proline in the conscious animal. Therefore, the examination using the immunohistochemical double-labeling method in the conscious animal was fit to examine responding neurons to L-proline under the conscious state.

### 3.1. Cardiovascular Responses

**Figure 1:**
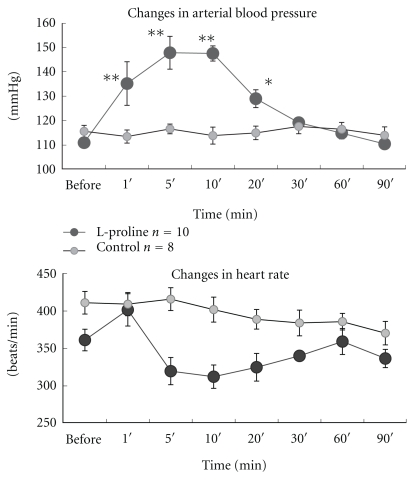
Changes in arterial blood pressure and heart rate following intracisternal injection of L-proline or ACSF. The arterial blood pressure increased, but the mean heart rate decreased, 5–30 min after an intracisternal injection of L-proline in freely moving rats. One-way ANOVA showed a significant difference (*P* < 0.005) in the heart rate of the L-proline-treated group, but no specific times were detected by Dunnett's test when control value was “before.” Results are presented as means ± SE. **P < 0.01 and *P < 0.05 by one-way ANOVA followed by Dunnett's test.

The changes in mean arterial blood pressure and heart rate of 10 L-proline-injected rats and eight ACSF-injected control rats are shown in Figure 1. Intracisternal injection of L-proline (1 M, 10 *μ*L) produced pressor and bradycardic responses, as observed in previous studies [4, 11]. The previous study using sinoaortic denervated rats [11] also suggested that bradycardic response is produced by the baroreceptor reflex as a secondary effect of the pressor action of L-proline stimulation. 

Some might think the hyperosmotic concentration of L-proline injected into the cisterna magna (1 M, 10 *μ*L) but not L-proline itself could produce hyperosmotic effect on osmosensitive structures in the hypothalamus to release vasopressin into the blood, resulting in the pressor response. However, hypertonic mannitol injections (1 M, 20 *μ*L) into the same cisterna magna of the conscious rat produced no change in blood pressure [22], and D-proline (1 M, 10 *μ*L) rather produced a depressor or no response [11]. It appears to become osmoinsensitive concentration when the solution reaches the osmo-sensitive structures along the flow path, possibly due to diffusion and constant flow of cerebrospinal fluid. In addition, the pressor response was completely blocked with kynurenate, the antagonist for ionotropic excitatory amino acid receptors [5]. Therefore, L-proline itself would be responsible for the pressor response, through activation of the L-proline-sensitive ionotropic receptors, but not of the osmotic receptors for general hypertonic solutions.

### 3.2. Double-Labeling of Hypothalamic Nuclei by Immunohistochemistry

**Figure 2:**
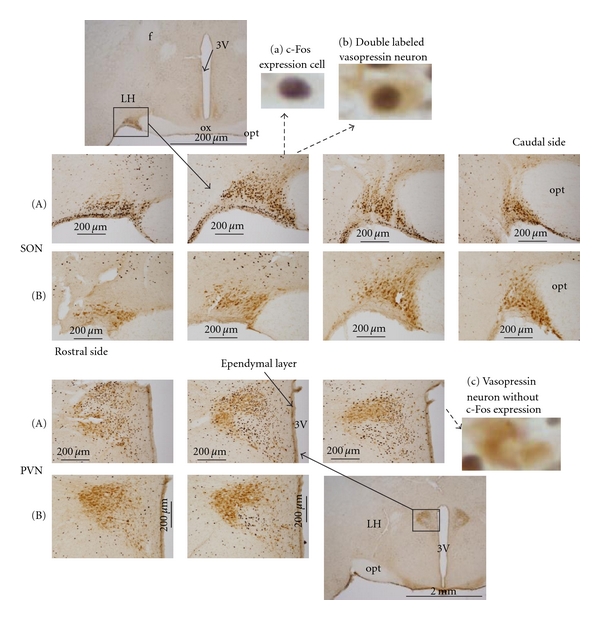
Frontal sections showing vasopressin-expressing neurons in the hypothalamic SON and PVN. (A) Intracisternal L-proline-injected rats. (B) Negative control, ACSF-injected rats. Representative higher magnification photographs are shown in (a), (b), and (c). The arrows indicate the location of the magnification in the original photograph. f: fornix, 3V: third ventricle, OX: optic chiasm, opt: optic tract, LH: lateral hypothalamic area.

**Figure 3:**
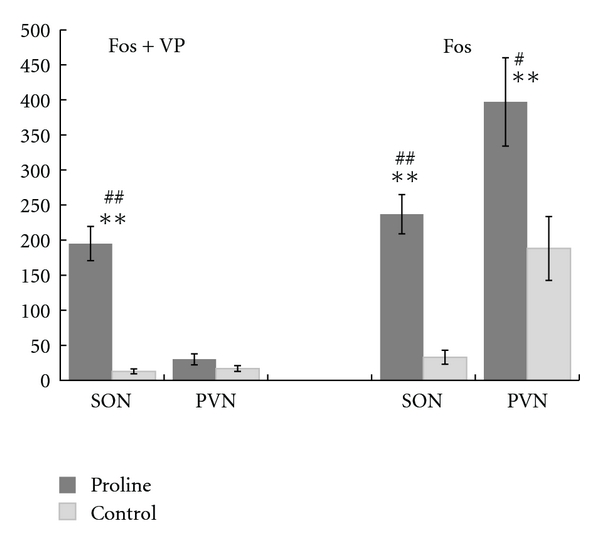
c-Fos-positive and vasopressin-positive neurons in the SON and the PVN following L-proline or ACSF injection. There were 10 L-proline-injected samples and eight negative control, ACSF-injected samples. “Fos + VP” indicates the number of vasopressin-expressing (VP) neurons with c-Fos stained nuclei. “Fos” indicates the total number of neurons with c-Fos stained nuclei. Data are presented as the means ± SE. **P < 0.01 by *t*-test. ^#^ *P* < 0.05 and ^##^ *P* < 0.01 by the Wilcoxon rank sum non-parametric test.

Brain sections of vasopressin-expressing neurons in L-proline-injected and ACSF-injected rats are shown in Figure 2. Activated neurons were marked by black material in their nuclei, which represents c-Fos protein expression (Figure 2A (a)). Activation of a vasopressin-expressing neuron was indicated by a black nucleus surrounded by a brown stain, as shown in Figure 2A (b). A vasopressin-expressing neuron that was not activated is shown in Figure 2A (c) for comparison. In L-proline-injected rats, sections at every level of the SON contained a high number of activated vasopressin-expressing neurons, but sections of the PVN had fewer activated vasopressin-expressing neurons (Figure 2A). Sections from the ACSF-injected rats (negative controls) showed a lower number of activated vasopressin-expressing neurons than the L-proline-injected rats in both the SON and the PVN (Figure 2B). In two pilot experiments using D-proline (1 M, 10 *μ*L), there were a few activated vasopressin-expressing neurons in both the SON and PVN, similar to the ACSF-injected rats. Vasopressin-expressing neurons were selectively activated in the SON, but not in the PVN, in L-proline-injected rats, as summarized in Figure 3 (left panel). 

To activate vasopressin neurons only in the SON with intracisternally injected L-proline, direct stimulation of the SON neurons would be possible. In the previous study, the cardiovascular responses to intracisternally injected L-proline were almost completely inhibited by a previous injection of the broad type ionotropic excitatory amino acid receptors antagonist [5]. If L-proline directly stimulates the vasopressin neurons in the SON, it would be through the ionotropic excitatory amino acid receptors. Are there the possible receptors in vasopressin neurons of the hypothalamus? Microinjection studies using the well-established endogenous excitatory amino acid receptor agonist L-glutamate combined with pharmacological methods suggest mediation of the non-NMDA ionotropic excitatory amino acid receptor but not of the NMDA receptor for vasopressin release in the neurons of the SON [23] as well as the PVN [24], monitoring blood pressure and heart rate in conscious rats. In the current study, two rats showed back flow of dye into the third ventricle close to the PVN, as mentioned later, but no significant staining of vasopressin neurons in the PVN. The reached concentration of L-proline might be too low to produce any response in the PVN. Microinjection experiments of L-proline itself into either nucleus of the hypothalamus could be helpful to understand the exact action of L-proline in the PVN as well as in the SON.

### 3.3. Unspecified Cells with c-Fos Expression in the Hypothalamus

In addition to vasopressin-expressing neurons, unspecified cells expressing c-Fos were also detected after L-proline stimulation, particularly in the PVN of the hypothalamus (Figure 3, right panel). In the SON, 80% of the c-Fos-positive cells were vasopressin-expressing neurons. With respect to the large number of unspecified, activated cells in the PVN, widely connected inputs expressing a broad range of neurotransmitters have been described in the brainstem and forebrain, including the hypothalamus [14]. The L-proline solution flowing from the cisterna magna may contact several loci, including A1 and A2 neurons in the medullary surface and the arcuate nucleus in the hypothalamus. There are numerous possible active sites and/or pathways linking L-proline to the PVN, and the underlying causes remain elusive. L-Proline appears to activate neurons in the brain other than supraoptic vasopressin-expressing neurons. 

Based on the results of a preliminary study [15], unspecified c-Fos was predicted to be expressed in vasopressin-containing neurons in both the SON and the PVN, because both nuclei showed high levels of c-Fos expression that coincided with changes in blood pressure. The preliminary results [15] were in agreement with pharmacological evidence showing that an intravenous injection of a vasopressin V1 receptor blocker effectively inhibited the hypertensive response to L-proline [11]. However, brain regions containing vasopressin-expressing neurons also contain neurons expressing a wide variety of neurotransmitters [14]. Therefore, the current double-labeling analyses were performed to determine whether intracisternal injection of L-proline activated vasopressin-expressing neurons in the hypothalamus to produce hypertension. Unexpectedly, vasopressin-containing neurons were selectively activated only in the SON, but unspecified neurons were activated in the PVN, after intracisternal stimulation by L-proline.

### 3.4. Dye Distribution

The previous pharmacological examination suggested a major involvement of vasopressin release in the L-proline-produced hypertension [11]. There could be direct contact of flowed solution from the cisterna magna to the vasopressin-containing neurons only in the SON located on the bottom of the cranial bone. Therefore, possible diffused areas were confirmed especially in the hypothalamus after dye injection. Evans blue solution was distributed in the SON on both sides of the optic chiasm at the base of the brain in all rats included in the current study. Two of the ten rats injected with L-proline exhibited backflow of the dye solution into the third ventricle close to the PVN. Dye also spread over the medullary surface and the caudal cerebellar surface facing the cisterna magna, as described previously [12, 13]. This dye distribution suggests a possible direct activation of the neurons in the SON by L-proline flowing from the cisterna magna and another possible indirect activation through the medullary noradrenergic A1 neuron.

### 3.5. Double-Labeling of Ventral Medullary Cells by Immunohistochemistry

**Figure 4:**
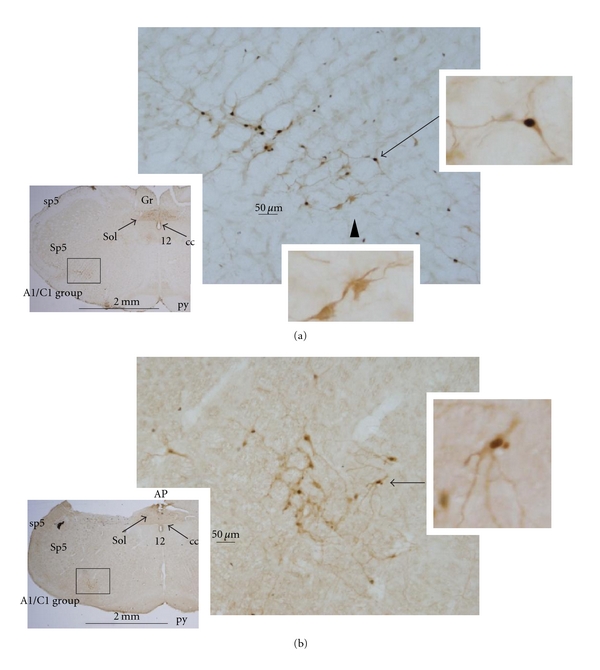
Double-labeled neurons showing black c-Fos nuclei and brown tyrosine hydroxylase-expressing (TH) neurons in medullary sections from (a) L-proline-injected rats, and (b) negative control, ACSF-injected rats. Arrows indicate double-stained TH neurons, and arrowheads alone indicate TH neurons without c-Fos expression, as shown in the magnified photographs. The framed regions in the small photographs (A1/C1 group) are shown magnified to the right. AP: area postrema, cc: central canal, Gr: gracile nucleus, py: pyramidal tract, Sol: nucleus solitary tract, sp5: spinal trigeminal tract, and Sp5: spinal trigeminal nucleus.

**Figure 5:**
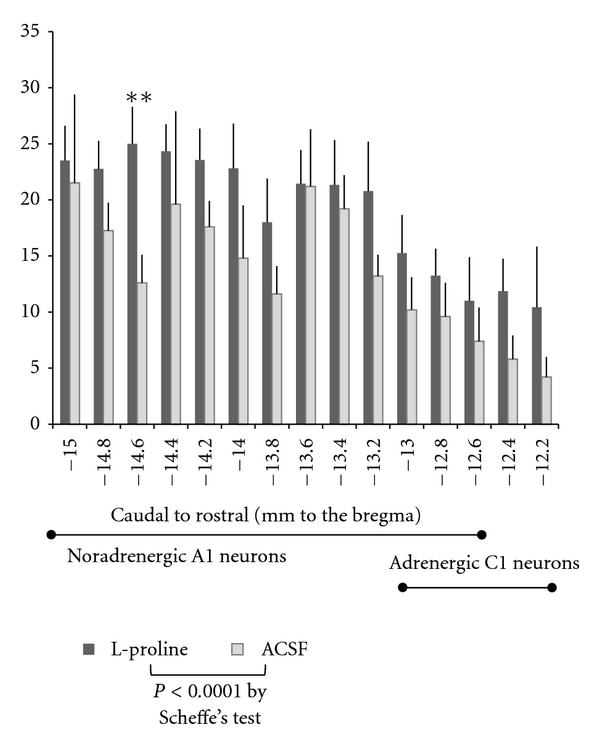
Numbers of c-Fos-positive and TH-positive neurons in the medulla. Data are represented as the means + SE. Repeated measures two-way ANOVAs indicated significant differences (*P* < 0.0003) between two drug treatments as well as among distance levels, and Scheffe's test supported the significant effect of L-proline but detected no significant levels among distance levels. On the basis of the hypothesis that the corresponding levels might have distinct sensitivities to L-proline, *t*-test between L-proline and ACSF detected significant difference at the slice level of −14.6mm (**P < 0.01). A1 or C1 is located in the part of bar, according to Buller et al. [18].

Medullary slices from both L-proline-injected and control rats showed high numbers of activated TH-expressing neurons, as shown in Figure 4. TH is a marker enzyme for catecholamine-containing A1/C1 cells. Figure 5 summarizes the numbers of double-labeled neurons in slices made across the ventral medulla every 0.2 mm, expressing the summed number of cells with two slices (100 *μ*m) in one distance level.

### 3.6. Data Analyses of Medullary TH-Containing Neurons

The data on double-labeling cells of c-Fos and TH were statistically analyzed. First, repeated measures two-way ANOVAs were performed in two factors of drug treatments and medullary distance levels. Thereafter, *t*-test was also applied to drug treatments at each medullary distance level based on a hypothesis.

There were significant differences in the number of double-labeled neurons between two treatments of L-proline and ACSF (*P* < 0.0003), as well as among slice levels (*P* < 0.0001) without interaction, according to repeated measures two-way ANOVAs. Comparisons of each level with Scheffe's test supported significant difference (*P* < 0.0001) between L-proline (mean ± SE, 19.2 ± 1.0, *n* = 114) and ACSF (13.5 ± 1.1, *n* = 73) but detected no significant difference among slice levels. The analyses indicate that L-proline significantly activates TH-containing A1/C1 neurons across the medulla, but with unspecified levels. 

Because sensitivities to L-proline stimulation remain unknown at each TH-expressing neuron, some TH-expressing neurons might respond to L-proline and the other might not respond. Therefore, *t*-test was applied to each combination at different distance levels, on the basis of the hypothesis that each population at different levels is independent. Only one level at −14.6 mm in Figure 5 (but four slices in the original counting number around this level) in the caudal medulla was detected with significant difference (*P* < 0.007). According to Buller et al. [18], the levels between −15 mm and −13.2 mm from Bregma in Figure 5 are included only in the A1 group, and these levels contained the slices of significantly activated TH-containing neurons following L-proline stimulation. Several specific caudal A1 neurons may be highly sensitive to L-proline stimulations. 

With respect to the potential activation of the pathway from A1 to the SON, the current immunohistochemical data and analyses did not give convincing support, although neurons across A1/C1 areas were significantly activated by L-proline stimulation. Additional investigations are required for understanding of the network activation with L-proline stimulation in details.

## 4. Conclusion

The current immunohistochemical double-labeling study clearly demonstrated that intracisternal injection of the neurotransmitter candidate L-proline produces preferentially strong activation of vasopressin-expressing neurons only in the SON, but not in the PVN, in freely moving rats. The other neurons including ventral medullary TH (A1/C1) neurons and neurons other than vasopressin-expressing neurons in the PVN were also activated by intracisternally injected L-proline. The data could give a clue to find acting sites of L-proline in the brain of freely moving rats.
